# Nailfold Capillaroscopy in Rheumatic Diseases: Which Parameters Should Be Evaluated?

**DOI:** 10.1155/2015/974530

**Published:** 2015-09-01

**Authors:** Mahnaz Etehad Tavakol, Alimohammad Fatemi, Abdolamir Karbalaie, Zahra Emrani, Björn-Erik Erlandsson

**Affiliations:** ^1^Medical Image and Signal Processing Research Center, Isfahan University of Medical Sciences, Isfahan 81745-319, Iran; ^2^Department of Rheumatology, Alzahra Hospital, Isfahan University of Medical Sciences, Isfahan 8174675731, Iran; ^3^School of Technology and Health (STH), Royal Institute of Technology (KTH), 141 52 Huddinge, Sweden

## Abstract

Video nailfold capillaroscopy (NFC), considered as an extension of the widefield technique, allows a more accurate measuring and storing of capillary data and a better defining, analyzing, and quantifying of capillary abnormalities. Capillaroscopic study is often performed on the patients suspected of having microcirculation problems such as Raynaud's phenomenon as the main indication for nailfold capillaroscopy. Capillaroscopic findings based on microcirculation studies can provide useful information in the fields of pathophysiology, differential diagnosis, and monitoring therapy. Nailfold capillaroscopy provides a vital assessment in clinical practices and research; for example, its reputation in the early diagnosis of systemic sclerosis is well established and it is also used as a classification criterion in this regard. This review focuses on the manner of performing video nailfold capillaroscopy and on a common approach for measuring capillary dimensions in fingers and toes.

## 1. Introduction

Nailfold capillaroscopy is a highly sensitive, inexpensive, simple, safe, and noninvasive imaging technique used in the morphological analysis of nourishing capillaries in the nailfold area [1].

The early detection of microvascular changes that can occur in some inflammatory connective tissue diseases is the main advantage of capillaroscopy, which has attracted the attention of many rheumatologists. Recently, in the American College of Rheumatology/European League Against Rheumatism, this feature of capillaroscopy has been suggested as an additional criterion for the preliminary classification of systemic sclerosis (SSc) [2].

When used together, autoantibodies and capillaroscopic findings are generally accepted as a powerful diagnostic tool for detecting emerging connective tissue diseases (CTDs) in patients with Raynaud's phenomenon [3–7]. Different parameters such as antinuclear antibodies (ANA), number of capillaries, and the presence of giant capillaries have been combined in an algorithm called PRINCESS (Prognostic Rule-Based Instructions using Nailfold Capillaroscopy Examination and Scleroderma-Related Serology). This has allowed the stratification of Raynaud's phenomenon (RP) based on the SSc incident risk [4, 8].

Nowadays, this method is used for a group of scleroderma-spectrum disorders to differentiate the primary Raynaud's phenomenon (RP) from the secondary RP in rheumatic diseases. Analogous changes may be observed in other connective tissue diseases (CTDs) such as mixed connective tissue disease, overlap syndromes, dermatomyositis, and polymyositis.

## 2. Search Strategy

Studies published from January 1990 up to November 2014 have been collected in “PubMed” and “Embase” databases by employing a systematic literature search. In addition, the bibliographies of these articles and the previously published reviews have been manually searched to help augment the process. The repeated references have been excluded. All the relevant literatures in English and German languages have been searched. Boolean operators “AND” and “OR” together with keywords such as “capillaroscopy,” “nailfold capillaroscopy,” “video nailfold capillaroscopy,” “nailfold video capillaroscopy,” and “Kapillarmikroskopie” have been used to make the search more specific and to reduce the sensitivity of the search.

The search results have been reviewed and discussed by two independent reviewers. The full texts of articles that seemed to comply with the set of criteria in this study were obtained and checked for relevancy by the reviewers. The discrepancies between the abstract and other parts of a paper were discussed as necessary, and a discretionary decision was made as to include or exclude a particular article.

## 3. Capillaroscopy Procedures

Surprisingly, the capillaries in the identical fingers of an individual have the same morphological patterns, and regardless of the age, in healthy subjects, these patterns are similar [9]. In most areas of the fingers, the nutritional capillary loops are oriented at 90° to the skin surface. Only the tips of the capillary loops can be observed. Usually, there are one to three capillaries in each dermal papilla. The capillary loops become more parallel to the skin surface in the nailfold areas. They are observable in their full length in the last row [10].

Although nailfold capillary blood flow may slow down in normal subjects exposed to cold, it does not come to a complete standstill even after an exposure of more than 30 minutes. In certain stages of the disease, severely slow capillary flow occurs in nailfold loops within a matter of minutes and the flow stops quickly in all the end-row loops and beyond. Investigators have specified a temperature range of 10–20°C for this condition [11].

Various devices can be used for capillaroscopic analysis: the wide field microscope, the dermatoscope, the videocapillaroscope, and the ophthalmoscope. A digital videocapillaroscope combines a microscope with a digital video camera and it is considered as the main tool for measuring and evaluating capillaroscopic parameters. It has a low magnification capability but can also take advantage of its sequential high magnification function to distinguish the capillaries based on observable details. Furthermore, it allows a direct contact with a nailfold and also enables the examination of patients with severe finger flexion contractures [12].

By employing the software integrated with an image digitizer board, the acquired images can be processed. The program takes full advantage of the quality and format characteristics of the multiple video outputs of videocapillaroscopy by using a developed board technology. Although this program is extremely easy and flexible to use, it is highly sophisticated. It can be used for research purposes as well as clinical practice, providing rapid information retrieval and the visualization of previously acquired images. Digital filters such as grey scale, watershed, and top hat filters can be applied to separate the relevant areas from image background. The filters can also be applied in succession. Extremely precise measurements can be achieved by using the software and the image magnification capability. Specifically, length, diameter, area, and density can be measured, and then these measurements can be stored in the image [13].

There are no limitations on the number of fingers or the number of fields chosen for examination. Some authors have chosen the fourth and fifth fingers of both hands [14], others have chosen all the fingers of both hands except the thumbs [15, 16], and many have worked with the whole fingers of both hands [17–21]. The capillaroscopy procedure will be explained in the following section.

**Figure 1:**
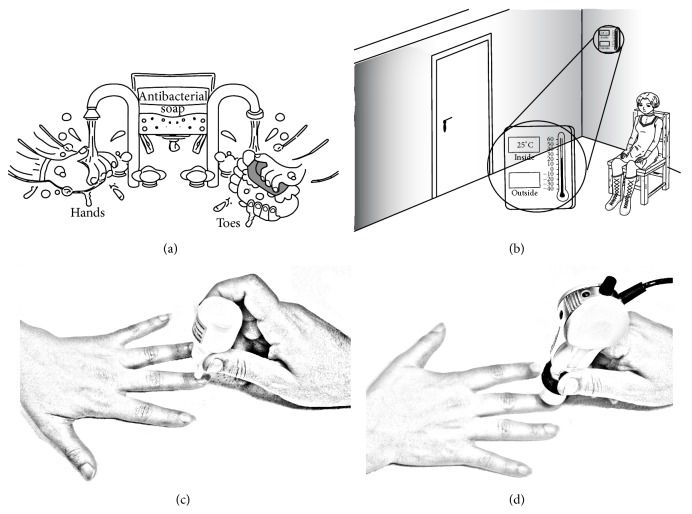
Various steps required for a nailfold capillaroscopy procedure.

*Washing the Hands or Toes*. Fingers and toes must be clean. In some cases, they are not sufficiently clean. The examiner will have the patients wash their hands or toes gently with antibacterial soap and water. They may lightly wipe them with an alcohol sponge [22] (Figure 1(a)). 

*Acclimatization.* In order to acclimatize the patients and get them relaxed before the test, they should be seated at room temperature (20–25°C) for 15–20 minutes. Then, depending on the outside temperature, a patient's hands would be positioned at her heart level [14, 23–25] (Figure 1(b)).

*Improving the Visibility of Capillaries*. In order to improve the image resolution, a drop of vegetable oil is placed on the nailfold of each finger or toe before observation [26] (Figure 1(c)).

*Contact of Videocapillaroscope*. A videocapillaroscope directly contacts a patient's nailfold. In order to minimize the reflections, the contact angle and direction of the videocapillaroscope may be changed. A sharp image of capillary branches can be acquired by manually adjusting the focusing system and using the camera head [27] (Figure 1(d)).

*Image Capturing.* Four consecutive images (1 × 1 mm in size) are usually taken from the middle of a nailfold by a videocapillaroscope at a magnification of 200x [26, 28].

*Capillary Patterns Study*. After evaluating the patients with different microvascular abnormalities, an experienced observer compares and discusses the stored images of capillary patterns [29].

*Important Points and Considerations to Note in a Capillaroscopy Procedure*

- The patients are asked to avoid taking caffeine and smoking for 4–6 hours before the examination [30].
- The patients are instructed not to remove their fingernail cuticles for one month to avoid microtraumas that could put the examination at risk [31].
- Only vegetable oils (neutral oils) that are skin-friendly, such as walnut oil, cedar wood oil, olive oil, and peanut oil, should be used in the procedure. Applying common immersion oils used in microscopy may cause skin and mucous membrane irritation [32].
- Physically injured fingers are excluded from the study [29].
- Before the capillaroscopy of toes, the patients are asked to use a soft brush to clean their toenails and remove any dirt and bacteria that may get stuck in the nail corners, while taking care not to damage the surrounding skin [33].
- It is better to set the capillaroscope on the floor during the examination of toes, but for a comparative examination of fingers, it is more appropriate to have the device on a table [34].
- The magnifying power of optical probes can range from 100x to 1000x, although a magnification of 200x is routinely employed in clinical practice. Capillaries can be observed more clearly at this magnification; however, only a quarter of the nailfold area can be covered by each capillaroscopic image produced in this way. At magnifications higher than 600x, the blood cells inside the capillaries can be visualized [27, 35].
- Since the fourth and fifth fingers of both hands have the highest skin transparency, the most precise morphologic evaluations can be obtained from these fingers [36, 37].
- Before the capillaroscopy procedure, the functions of the equipment may be explained to the patients, especially the children [20].
- Vasodilatation can be prevented by a cold light source. When using contact optical probes, even the application of minimal pressure on the nailfolds should be avoided, because this can modify the vessels [38].
- In order to properly observe all the morphostructural characteristics of the examined field, the capillaroscopy probe must be moved in a slow uniform motion [39].
- It is suggested that the images acquired during examination should be analyzed by two different experts with no prior knowledge of a patient's clinical conditions, because the measuring of capillary dimensions by hand is tedious and prone to inaccuracy [40].
- In the same finger of a healthy subject, the morphologic pattern of nailfold microcirculation remains usually constant for many years [9, 41].
- For a better imaging resolution, adding more oil could be beneficial. However, both too few or much oil could decrease resolution and should be avoided [42].
- It is quite common to have little or no capillary flow when the examination room is cool or the subject is nervous [42, 43].
- For each subject, the procedure takes about 15–30 minutes [44].

## 4. Number of Images Taken from Each Finger

One still image and a ten-second video film are recorded at the midline of a nailfold after focusing the instrument. The target area for video recording is the distal layer of the capillaries under the papilla. A few functioning capillaries with flowing red cells are included in the recorded film. A series of partially overlapping images (about 4–12 images) are taken from the very left to the right to cover the whole nailfold.

**Figure 2:**
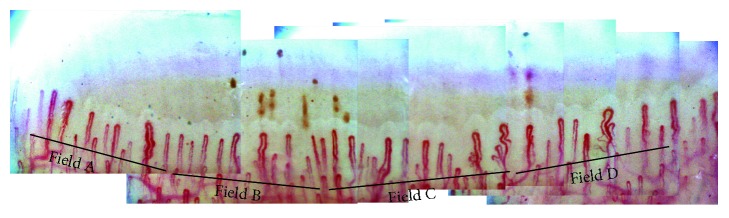
Panoramic mosaic image at 200x magnification.

A window of the same pattern is made on the first and second images. Then by calculating the areas of highest correlation, the two images are joined together. This procedure is repeated to produce a full composite image. In this case, a panoramic video of the nailfold with the manual production of a single panoramic mosaic of each nailfold is provided [24, 45] (see Figure 2).

*Note.* The number of produced images depends on the magnification of the videocapillaroscope.

## 5. Capillary Density

Capillary density is one of the most important parameters for the early diagnosis of individuals with an underlying risk for various connective tissue diseases [17]. Ingegnoli et al. [4] designed a prognostic model that can be used to facilitate clinical decision making in the screening phase. In this model, capillary density, which is in the form of an index, can characterize the potential patients better than the prognostic index. In addition, this parameter plays a fundamental role in the calculation of capillaroscopic skin ulcer risk index (CSURI) [23].

 Capillary density is defined as the number of capillaries in a 1 mm length of the distal row of each finger or toe. Capillary density is also known as the “number of capillaries” and “capillaries number.”

Some researchers consider toe capillaroscopy as an unreliable tool in clinical practice [31]. For this reason, few studies have been carried out in this field. The capillary density of toes seems to be different in healthy adults across Europe. For example, German authors reported a mean capillary density of 6.6 ± 0.97 (mean ± standard deviation (SD)) with a range of 5–9 capillaries per millimeter [34]. On the other hand, a Bulgarian group showed slightly higher capillary counts than those of the German authors. They obtained an average capillary density of 10 ± 0.59 capillaries per millimeter.

Nailfold capillary density appears to be similar in healthy adults and healthy children across Europe. European authors found the mean capillary density in healthy children to be in the range of 5–7.3 compared to 7.3–10.3 in healthy adults. Brazilian authors showed slightly higher capillary counts, ranging from 6–7.3 capillaries per millimeter in children to 9.11–10.1 capillaries per millimeter in adults.

### 5.1. Counting Number of Capillaries

**Figure 3:**
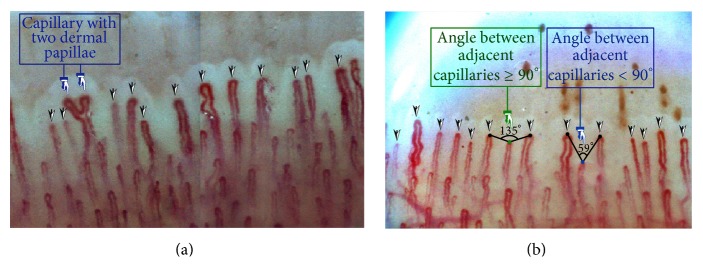
(a) Measurement of capillary density by the direct observation method and (b) measurement of capillary density by the 90° method.

Several methods have been provided for calculating the capillary density [23, 46]. In 2009, Sebastiani et al. [23] used a specific method for counting the number of capillaries for CSURI index. In this approach, all the capillaries present in the distal row are considered for counting, even if they are not at the same levels. In case a ramified capillary occupies more than one dermal papilla, the number of papillae is counted (Figure 3(a)).

In the cases in which the capillary density is high, it is difficult to tell which capillaries belong to the distal row. Hofstee et al. [46] proposed two methods for estimating the capillary density, with the first one being the direct observation method. In this technique the capillary loops are observed directly, and those considered to be distal loops are marked. In the second method, called the 90° method, a capillary loop will be considered as a distal loop if the angle between the apex of that capillary and the apex of its two adjacent capillaries is greater than 90° (Figure 3(b)). Since there is no standardized approach to measure the capillary density, most authors use the direct observation or the 90° method for measuring the number of capillaries. Figures 3(a) and 3(b) illustrate the direct observation and the 90° methods, respectively.

### 5.2. Scoring System of Capillary Density

Some investigators have introduced various scoring systems to categorize capillaroscopy findings in healthy individuals. Ingegnoli et al. [17] classified the numbers of capillaries counted in a 1 × 1 mm area based on a scoring system using ordinal numbers. Also, Hoerth et al. [29] developed a scoring system based on age as an effective parameter to count and categorize the numbers of capillaries (Table 1). Cutolo and Smith [28] have proposed that the number of abnormal capillaries (parameters) should be based on the number of capillaries counted along one linear millimeter of a nailfold's distal row. The mean score for each capillaroscopic parameter should be obtained by analyzing at least two or four fields in the middle region of the nailfold in each finger. The average scores from the fields of each of the eight fingers are summed up and then the total value is divided by eight. The resulting value is the score for each analyzed capillaroscopic parameter (0–3).

*Important Observations Related to Capillary Density*

- Hoerth et al. [29] and Dolezalova et al. have demonstrated that capillary density is directly related to age. Younger children have fewer capillaries than older children and adults.
- Gender differences do not have a large impact on the number of capillaries [29].
- Capillaroscopy is possible for skins with light pigmentation and is difficult to perform on skins with dark pigmentation [47].
- In countries with different ethnic groups, it is preferred to classify the patients as whites and nonwhites. According to [48], capillary density in both the white and nonwhite groups increases progressively with age.
- Fingers have a higher capillary density than toes [34].
- It is advised to calculate the mean capillary density value for each finger from the analysis of four fields on that finger. The mean value is obtained from the number of capillaries in the tested finger [28], as is shown in Figure 3.

## 6. Measurement of Capillary Dimensions

Some important capillary dimensions are introduced in this section. These dimensions include the capillary width, capillary length, arterial limb diameter, venous limb diameter, external diameter, internal diameter, and the apex width. By using the available software programs, some of these dimensions such as capillary width, capillary length, and intercapillary distance can be measured quantitatively.

The width and height of a capillary normally indicate the health condition of a person. An individual with an elongated capillary usually suffers from hypertension and arteriosclerosis, while a shorter capillary often points to cardiac insufficiency [49].

Different values have been reported in the literature for the capillary width parameter. Some studies have reported the widths of the arterial, the venous capillary limb, and the apical capillary loop, while others have used the whole width of the capillary loop. In the following, all the capillary dimensions are described separately for a better understanding of capillary structure.

**Figure 4:**
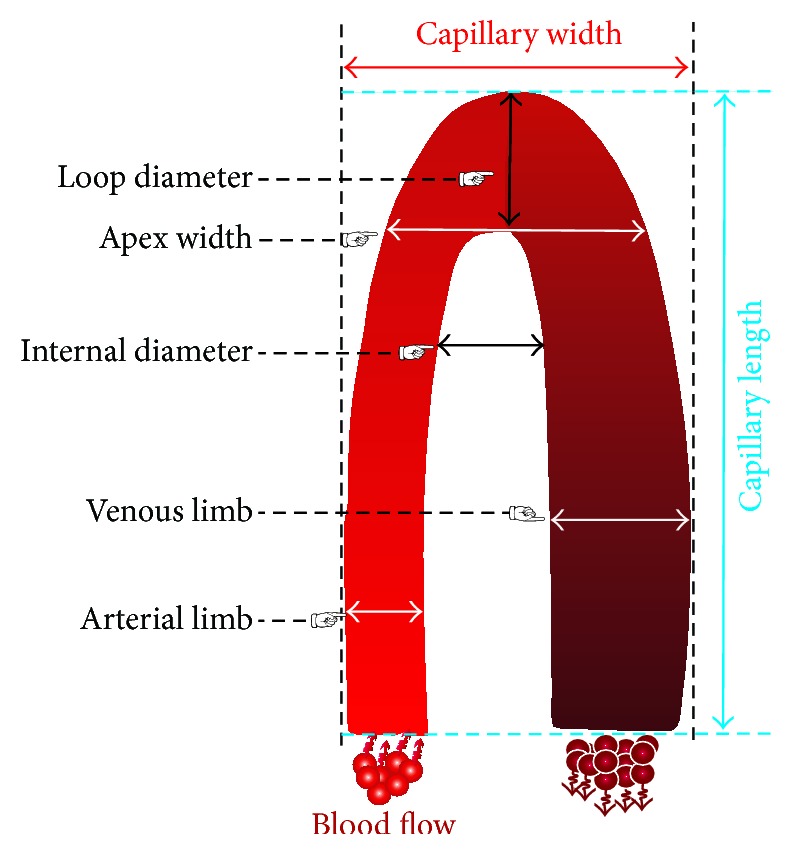
Schematic drawing of the front portion of a nailfold capillary loop.

A schematic drawing of the front portion of a nailfold capillary loop is shown in Figure 4, and some capillaroscopic terms in this figure are explained below.

### 6.1. Capillary Width (CW)

Capillary width is expressed as CW = *X*_Rmost_ − *X*_Lmost_, where *X*_Rmost_ and *X*_Lmost_ are, respectively, the *x*-coordinates of the rightmost and leftmost points, shown in Figures 6(a) and 6(b) [49]. Based on Table 3, the normal upper limit for the total capillary width is 27–59.5 *μ*m and its mean value is 44 ± 4.8 *μ*m (mean ± SD).

 Capillary width (CW) is the width of a capillary loop at its widest section [14, 17, 44, 50]. Capillary width is also known as “loop width,” “maximum loop width,” “total width,” “total caliber of loop,” “capillary loop amplitude,” “total capillary width,” and “external diameter.”

#### 6.1.1. Enlarged Capillary

Capillary width is a controversial parameter for scoring, because various researchers define and score it in different ways, and there is no general consensus. Some authors have based their rating system on Maricq's work, which defines capillary enlargement as a 4- to 10-fold increase in capillary size [25]. In most studies, enlarged capillary has been defined as follows:

 The capillary width of enlarged capillaries is at least 90 to 150 *μ*m (0.090 to 0.150 mm) [25, 41, 51–55]. An enlarged capillary is also known as “loop enlargement” and “enlarged loop.”

**Figure 5:**
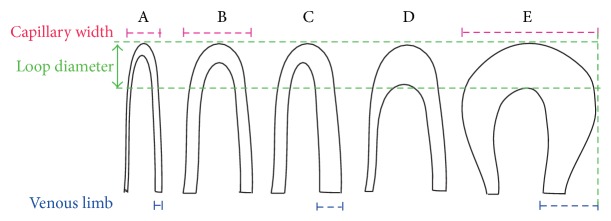
Enlarged capillary loops; (A) normal capillary loop, (B) enlarged afferent capillary loop, (C) enlarged efferent capillary loop, (D) enlarged apical capillary loop, and (E) horseshoe shape giant capillary loop.

Some researchers designate capillaries with a width of at least 50 *μ*m (0.050 mm) [56, 57] as enlarged capillaries or present capillary sizes with regard to individual limb widths [44, 58–60] or capillary loop areas. In adults, the upper limit of a normal capillary loop area ranges from 25 to 50 *μ*m (0.025 to 0.050 mm) [25]. The three types of enlarged capillary loops are the enlarged afferent, enlarged efferent, and enlarged apical capillaries, shown in Figures 5(B), 5(C), and 5(D) [61]. Capillary enlargement occurs in many conditions; however, homogeneously enlarged capillaries are typical of three connective tissue diseases: scleroderma, mixed connective tissue disease, and dermatomyositis [62].

#### 6.1.2. Enlarged Capillary Scoring System

Some investigators have presented several scoring systems for categorizing capillaroscopy findings in healthy individuals. Koenig et al. [3] and Dinç et al. [63] have proposed a scoring method based on ordinal numbers to assess capillary enlargement.  Table 2 shows the scoring system for enlarged capillaries.

### 6.2. Capillary Length (CL)

**Figure 6:**
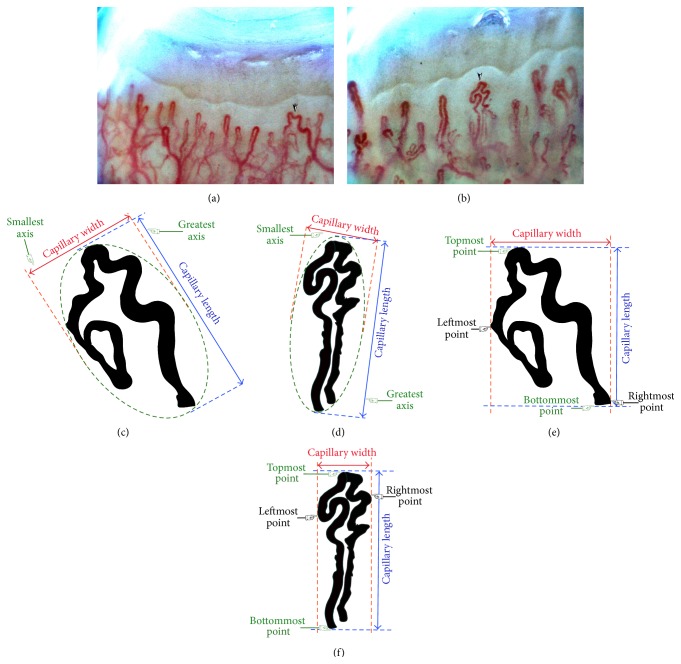
(a, b) Two different images acquired by a digital capillaroscope with 200x magnification from two young healthy females to extract the features of capillaries marked with arrows. (c, d) Automated measuring of capillary width and height (PCA method). (e, f) Manual measuring of capillary width and height.

Capillary length is expressed as CL = *Y*_Bmost_ − *Y*_Tmost_, where *Y*_Bmost_ and *Y*_Tmost_ denote the *y*-coordinates of the bottommost and topmost points, respectively, as shown in Figures 6(a) and 6(b) [49]. According to Table 3, the normal upper limit for the whole capillary length is between 92 and 295 *μ*m and its mean value is 240 ± 38.3 *μ*m (mean ± SD).

 Capillary length (CL) is the distance between the apex of a capillary loop and the point where the capillary loop is no longer visible. Capillary length is also known as “loop length” and “capillary height.”

#### 6.2.1. Elongated Capillary

Physiologically, capillaries in a person's identical fingers have a similar length, which varies from person to person and in physically altered fingers. An example would be fingers with a high nail wall or with a loss of extension of the finger shortening by a different angle of view. Diabetes patients often have shorter capillaries, sometimes only up to 10 *μ*m long (“shoal of fishes or elephant nose”) [32, 64]. By increasing this parameter, a special form of capillaries appears, which will be described in the following section.

 Capillary loops longer than 300 *μ*m (0.300 mm) are classified as elongated [36].

#### 6.2.2. Methods of Measuring Capillary Width and Length

Capillary width and length can be measured either manually or by an automated mechanism.

*Automatic Measurement*. The lengths of the major and minor axes of the ellipse obtained by the principal components analysis (PCA) can be used to measure the capillary width and height. This is illustrated in Figures 6(c) and 6(d). Hence, capillary height has twice the largest proper value, while capillary width has twice the smallest proper value [65].

*Manual Measurement*. In manual measurement, shown in Figures 6(e) and 6(f), capillary width is defined as the diameter of the arterial and venous limbs at their widest points. Also, capillary length is the distance between the bend of the capillary loop and its base. Sometimes it is difficult to define the latter mark, because it varies with the skin transparency of various subjects [66].

### 6.3. Arterial and Venous Limbs Diameters

It is well known that the venous limb (VL) refers to the efferent branch, while the arterial limb (AL) refers to the afferent branch of a capillary loop.

 The diameters of the arterial and venous limbs at their widest sections are considered as the arterial limb diameter and venous limb diameter [67]. Arterial and venous limb diameters are also called the “efferent limb width” and “afferent limb width,” respectively.

According to the values reported in Table 3, the arterial limb capillary diameter varies from 7 to 17 *μ*m and its mean value is 11.91 ± 1.87 *μ*m (mean ± SD). The upper limit of normal venous limb diameter in adults ranges from 11 to 20.6 *μ*m and its mean value is 15 ± 2.42 *μ*m (mean ± SD). A thinner arterial limb, a wider venous limb, and a connecting part (an apical loop) are present in every single capillary loop.

#### 6.3.1. Giant and Dilated Capillaries

Maricq et al. have designated as giant capillaries those vessels whose maximum widths are 4 to 10 times the maximum widths of normal vessels [25, 44, 66]. The presence of giant capillaries is the earliest and most striking sign of secondary Raynaud's phenomenon due to systemic sclerosis (SSc) [28].  Figure 5(E) shows a giant capillary loop with the diameter of its apical limb (loop diameter) being larger than the other two limbs.

- Those microvessels of either the arterial or venous limb with a diameter greater than 50 *μ*m (0.050 mm) are classified as giant capillary loops [36]. A giant capillary (see Figure 5(E)) is also known as a “Megacapillary.”
- A capillary whose arterial limb diameter is larger than 15 *μ*m or whose venous limb is wider than 20 *μ*m is classified as a dilated capillary [66].

### 6.4. Internal Diameter (ID)

Based on the data in Table 3, the upper limit of the normal internal diameter in adults ranges from 18 to 21 *μ*m.

 Internal diameter (ID) is the distance between the afferent and efferent limbs measured at the same level of a capillary loop [68]. Internal diameter is also called “distance between limbs.”

### 6.5. Loop Diameter

The apical loop in the central region refers to the whole width of a capillary loop at its widest point (Figure 4). The normal upper limit of a whole loop diameter is reported to be between 8 and 21 *μ*m, with its mean value being 17.17 ± 2.12 *μ*m (mean ± SD).

 Loop diameter is expressed as the diameter at the apex of a capillary loop. Loop diameter is also called “apex,” “apical diameter,” “apical loop,” “apical loop diameter,” and “transitional.”

### 6.6. Apex Width

Although Lefford and Edwards [44] have suggested that an index derived from apex width and maximum limb width (maximum distance between arterial and venous limbs) can distinguish abnormal capillary loops from normal ones [68], very few research works have emphasized this parameter. According to Table 3, the upper limit of normal capillary apex width in healthy adults ranges from 26 to 39 *μ*m and its mean is obtained as 36.2 ± 9.19 *μ*m (mean ± SD).

 Apex width is defined as the maximum open space measured in the apex of a capillary.

Although, by definition, total capillary width could be measured for all the capillaries in the 1 mm length (otherwise, that capillary would not be “counted”), in some cases, the capillary length, apex width, arterial diameter, internal diameter, venous diameter, and loop diameters cannot be measured due to a capillary's indistinctiveness.

Hence, the number of capillaries whose entire series of dimensions are measurable is also documented for each individual. It is essential to measure the total capillary width for all the capillaries, not just those whose dimensions of capillary length, apex width, arterial diameter, internal diameter, venous diameter, and loop diameter are measurable. Otherwise, the total capillary width will be underestimated due to the distortion of capillary architecture in some patients. The most abnormal capillaries would be excluded from the analyses [69].

#### 6.6.1. Systems for Scoring Capillary Length and Widths

Some investigators have introduced their own scoring system to differentiate capillaroscopy findings in healthy individuals. Ingegnoli et al. [17] have proposed a scale based on ordinal numbers to evaluate the lengths of capillary loops and also capillary widths. The scoring systems introduced for the capillary loops and widths have been presented in Table 4.

*Important Notes on Capillary Dimensions*

(i) Adults and older children have narrower capillaries than younger children. Thus, capillary width is age related [25].
(ii) The mean value of the maximum diameters of the 3 widest capillaries in the 1 mm length is called the “mean capillary width” [17, 66].
(iii) Generally, the arterial limb is narrower than the venous one, with the diameter increasing from the proximal arteriolar to the distal venous side. The capillary diameter is indicated by the erythrocyte column [10].
(iv) Capillary length is determined by measuring the three most visible parts of a capillary relative to the venular plexus [17, 54].
(v) The mean diameter of the arterial limb or the venous limb is defined as the mean value of 3 arterial or venous limbs at their widest sections [36].
(vi) The capillary lengths of the fourth and fifth fingers are always longer than that of the other fingers [38, 70].
(vii) The basin area of a capillary is defined by CW × CL, where CW and CL are the capillary width and length, respectively. All the relevant definitions have been shown in Figure 6 [49].

## 7. Intercapillary Distance (ICD)

According to Table 3, intercapillary distance varies from 96 to 166 *μ*m and its mean value is obtained as 137 ± 12.84 *μ*m (mean ± SD).

 Intercapillary distance is defined as the longest distance that exists between two neighboring capillary loops [71]. Intercapillary distance is also known as “interpeak capillary.”

### 7.1. Measuring the Intercapillary Distance

Manual measurement and semiautomated measurement methods are the two approaches used to determine the intercapillary distance [30].

*Manual Measurement Approach*. The apex tip of each capillary in a nailfold image is marked by a vascular technician with a cursor via a user interface. Then, the intercapillary distances are manually measured by using the marked points. In summary, the following three steps are involved in the manual measurement of intercapillary distance.

1. A least squares second-order polynomial curve is fitted to the specified apices.
2. The projections of the points onto the obtained curve are determined.
3. The mean distance between adjacent points is measured.

**Figure 7:**
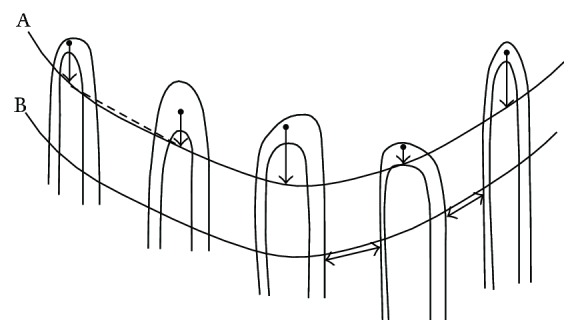
(A) Manual measuring of the intercapillary distance and (B) automated measuring of the intercapillary distance.

The initial rotation of a finger under the microscope as well as the nailfold curvature is also taken into consideration. The outcome of the manual measurement approach is shown in Figure 7(A).

*Semiautomated Measurement Approach*. In this method, noise may be removed indirectly during image processing and enhancement, and so the size of large dilated capillaries may be overestimated. This newly developed system has a number of limitations. The preprocessing and enhancement of images are biased toward more normal capillaries. During this process, noise may be removed indirectly and therefore the size of larger capillaries might be underestimated, larger capillaries might become distorted in images, and the vascular areas might be overestimated. By marking the centerlines of capillaries and measuring the distance [distances] between their intersections with a line parallel to the locus of the apices, the intercapillary distance is obtained. The outcome of this approach is illustrated in Figure 7(B).

### 7.2. Intercapillary Distance Scoring Systems

Several systems have been presented for the scoring and classifying of intercapillary distance parameter in healthy individuals. Ingegnoli et al. [17] have used a scoring system based on ordinal numbers to evaluate the various intercapillary distances. The outcome is shown in Table 5.

## 8. Avascular Areas

Avascular areas can be defined in several ways. From a practical point of view, it is important to differentiate between avascular areas and areas with low capillary density. So, it is necessary to agree on a definition for avascular areas [72]. This parameter is defined with and without the consideration of intercapillary distance.

 Avascular areas refer to distinct areas in the nailfold where two or more capillaries are missing, as compared to the areas of low capillary density in the rest of the row [10]. Avascular areas are also called “reduced density of capillaries,” “loss of capillaries,” “vascular deletion areas,” and “deletion.”

Avascular areas are also defined as the distance greater than 500 *μ*m between two adjacent capillary loops from the distal rows or distance greater than 300 *μ*m in the proximal area [67]. According to [73], 92% of patients with Wegener's granulomatosis (WG) and 22–67% of patients with a mix of scleroderma (PSS), systematic lupus erythematosus (SLE), and connective tissue disease (MCTD) had avascular areas. In contrast to the mentioned patients, the patients with nonimmune disorders did not have avascular areas. Avascular areas may be related to tissue hypoxia. Since avascular areas have also been reported in more progressive diseases, these areas have a prognostic value [38].

### 8.1. Avascular Areas Scoring Systems

Avascular areas may be single or confluent. A 4-step scheme proposed for evaluating the degree of capillarity [17, 48, 54, 74] is illustrated in Table 6. Anders et al. [75] and Hofstee et al. [46] have classified avascular areas to three degrees (shown in Table 6).

*Important Notes on Avascular Areas*

- Even when general visibility is low, avascular areas are still well recognized [51].
- Avascular areas are more frequent in women (10%) than in men (4%). This difference is statistically significant for both genders and for general visibility classes [51].
- Physiologically, not all the considered capillaries can be used in the evaluation, because some of them are invisible due to being only partially filled. In these cases, a prolonged observation can be useful, because, often, after a few seconds, those capillaries will be filled again [32].

## 9. Capillary Distribution

**Figure 8:**
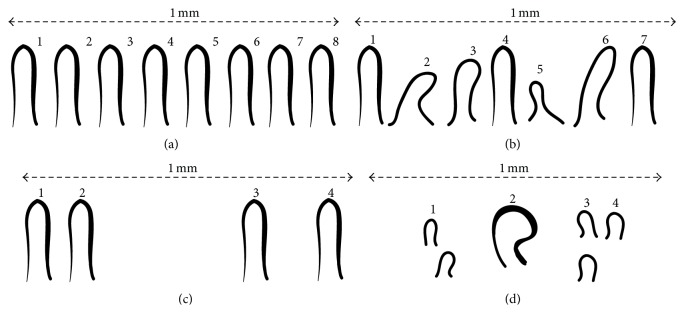
Distribution of capillary per mm; (a) normal capillary arrangement, (b) capillary disarrangement, (c) avascular areas (local paucity), and (d) an enlarged capillary and avascular areas [77].

In most areas of human body, capillary loops are perpendicular to the skin surface, whereas, in the nailfold region, they become parallel to the skin surface. A homogeneous and ordered distribution of capillaries arranged in parallel and at regular narrow distances between ascending and descending branches is found in capillaroscopy examinations of healthy subjects, including children and adolescents [76].  Figure 8(a) demonstrates the abovementioned features.

 Regular capillary distribution refers to a regular and orderly arrangement of capillaries in rows [77]. Capillary disorganization or disorganized architecture is defined as the complete distortion of a normal and regular capillary pattern.

### 9.1. Capillary Distribution Scoring Systems

Different capillary distributions are categorized by various scoring systems in order to differentiate capillaroscopy features in healthy individuals. These capillary distribution scoring systems have been introduced in Table 7.

*Important Notes on Capillary Distribution*

 The changes in normal capillary distribution are caused by the following characteristics of nailfold capillaroscopy: (a) capillaries that are not in one row, (b) small areas (<500 *μ*m) with missing capillaries existing next to areas with clusters of capillaries, (c) altered capillary distribution, (d) heterogeneity of loop shapes, and (e) irregular capillary orientation [38].

## 10. Capillary Shape

Although individual's capillaries have a tendency to become tortuous and dilated with age, their shapes may remain unchanged for many years. By applying a series of rapid control mechanisms including local metabolic and myogenic activity, optimal microvascular function can be maintained. As the microcirculation system remodels the blood vessels by changing their length, diameter, wall thickness, tortuosity, and number, permanent structural changes occur in these vessels over long periods. Although the possibility of obtaining more information from a quantitative approach is attractive, the visibility of these changes through capillary microscopy has prompted the use of a qualitative approach [61].

 A regular capillary is shaped like a hair pin or like the English letter “U” upside-down, with a slimmer arterial arm, an upper part, and a venous arm. The venous arm is larger than the upper part (as shown in Figure 4) [76].

### 10.1. Scoring System

The capillaroscopic patterns in SSc are specific. The presence of capillary shape (such as tortuous, branched, bushy, dilated, and giant capillaries), hemorrhages, elongated, capillary density and capillary arrangement are responsible for the classification. Ingegnoli et al. [20] developed a scoring system by combining quantitative and qualitative parameters (see Table 8). Their system is based on five qualitative and quantitative parameters: capillary density, capillary length, capillary shape, capillary arrangement, and pathologic hemorrhages. According to Table 8, the overall capillaroscopic patterns include the normal, minor abnormalities, major abnormalities, and the scleroderma pattern. In order to improve the diagnostic and prognostic power of capillaroscopic analysis, Pavlov-Dolijanovic et al. [78] proposed a classification method based on some selected characteristics of the disease progression. In patients with Raynaud's phenomenon, microvascular lesions detected by NVC have been reclassified into three different patterns: normal, nonspecific, and scleroderma like. In “normal” NVC pattern, typical hair pin structure or minor capillary morphological changes in distribution or size of loops are found. Moreover, “nonspecific” NVC pattern has the following features: meandering and crossed capillaries, capillary thinning, linear elongation of the loop, focal distribution of capillary hemorrhages, prominent subpapillary plexus, capillary spasm, nonhomogenous distribution or size of loops, widening of the afferent, apical, and efferent part of loop, and shortened loops. In addition, the NVC patterns are identified with the “scleroderma pattern” which in itself includes three patterns: “early” pattern that is described with few enlarged/giant capillaries, few capillary haemorrhages, no evident loss of capillaries, and relatively well-preserved capillary distribution; the second pattern or “active” pattern which has frequent giant capillaries, frequent capillary haemorrhages, moderate loss of capillaries, mild disorganisation of the capillary architecture, and absent or mild ramified capillaries; and the third pattern or “late” pattern, where irregular enlargement of the capillaries, few or absent giant capillaries and haemorrhages, disorganisation of the normal capillary array, severe loss of capillaries with extensive avascular areas, and ramified/bushy capillaries are identified as described by Cutolo et al. [74]. It is shown in Table 8.

## 11. Capillary Orientation

Capillary distribution is affected by the changes in the shape and direction of capillaries. Two parameters that can be useful in the investigation of capillary disorganization are capillary direction and capillary polarity.

**Figure 9:**
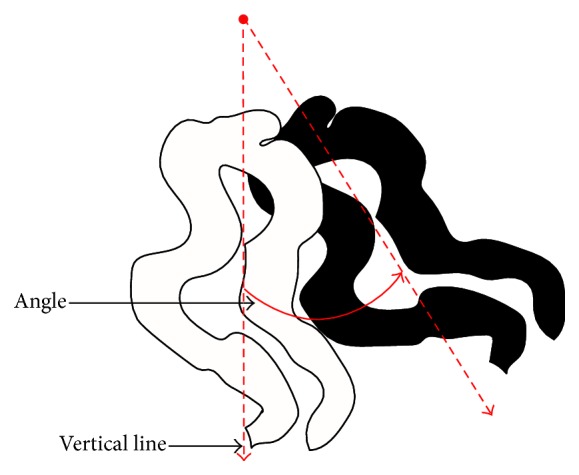
Automated PCA method for measuring the capillary orientation [65].

- Capillary direction is expressed as the angle between a vertical line and the vector associated with the highest proper value [65] (see Figure 9).
- Capillary polarity is defined as the standard deviation of all the capillary directions in an image [65].

## 12. Subpapillary Venous Plexus Visibility

Superficial vessels are connected to the deep arterial plexus and finally to terminal arterioles which make up the afferent limb of the capillary loops. These loops extend perpendicularly into the dermal papilla. The efferent limb incorporates into the superficial subpapillary venous plexus (PVS) and interfaces with the deep venous plexuses. Only the subpapillary venous plexus and the capillaries in the dermal papillae can be observed by the NFC [38].

 The vascular network at the base of a finger nailfold into which capillaries drain is called the subpapillary plexus.

Subpapillary plexus is normally observable at birth, but it becomes progressively invisible by adolescence. Only a few individuals have visible plexus after high school years [79–81]. The skin becomes more transparent in elderly people and the capillaries can be studied to a greater extent. The subpapillary venous plexus becomes clearly observable in some older patients [82].

According to a comparative study, blood flow in the subpapillary plexus is usually slower than that in the capillary loops. Even when blood flow slows down unusually, the subpapillary plexus is always clearly visible. In the case of decelerated blood flow, the condition is described as “dilated and prominent subpapillary plexus.” An evidence for decelerated blood flow is the dilation of the venous (efferent) limb of the capillary loop as well as a prominent subpapillary plexus [9, 67].

Although the underlying factor for plexus visibility is unknown, it is likely to be the thinness or transparency of the skin [78]. In patients with rheumatoid arthritis and also in children the subpapillary venous plexus is usually very prominent [26]. It is visible in approximately 30% of healthy individuals, more frequently in patients with systemic lupus erythematosus, and occasionally in people with systemic scleroderma [54, 76, 83].

### 12.1. Scoring System for Subpapillary Venous Plexus Visibility

The visibility of the subpapillary venous plexus was evaluated by the plexus visibility score (PVS) based on an ordinal scale proposed by Wertheimer and Werthelmer [84]. Depending on the extent of the distal phalange on which the plexus is visible, each finger is graded from 0 to 4. Consequently, the total score may range from 0 to 40. For practical purposes, PVS can be divided into 3 classes: low PVS (≤5), intermediate PVS (>5 and <10), and high PVS (>10) [48, 51]. The scoring systems for subpapillary venous plexus visibility are presented in Table 9.

*Important Points regarding the Subpapillary Venous Plexus*

- A comparative study showed that, statistically, the PVS for the left hand (1.49) is higher than that for the right hand (1.07). No other differences were discovered between the two hands [51].
- Usually, in patients with rheumatoid arthritis, and in some older patients as well as children, the subpapillary venous plexus is quite visible [26].
- In the nailfold capillaroscopy (NFC) procedure, only the subpapillary venous plexus and the capillaries in the dermal papillae are observable [38].

## 13. Conclusions

The simplicity, safety, and noninvasiveness of nailfold capillaroscopy are invaluable in detecting an underlying connective tissue disease (CTD) in patients with Raynaud's phenomenon (RP). Other novel treatments and specific therapies applied at an earlier prefibrotic stage of SSc may be more beneficial in mitigating and controlling the disease. However, in the progressive stages of the disease, capillaroscopy will be useful in monitoring the microvascular impact of drugs. Therefore, capillaroscopy can be considered as a fairly sensitive and highly specific test for detecting and screening scleroderma-spectrum disorders (SSDs) and a useful tool for monitoring and predicting organ involvement in scleroderma (SSc). This paper has presented a summary of the video nailfold capillaroscopy procedure and has reviewed the common techniques of measuring capillary dimensions and quantitative and qualitative methods of measuring morphological patterns [8].

## Figures and Tables

**Table 1:** Capillary density scoring systems.

Reference	Points	Capillary density per mm	
Lefford and Edwards [44] Ingegnoli et al. [17]	0	>9	
	1	>7–9	
	2	>4–7	
	3	⩽4	

Cutolo and Smith [28]	0	More than 9 capillaries	
	1	<33% reduction of capillaries (7–9 capillaries)	
	2	33–66% reduction of capillaries (4–6 capillaries)	
	3	>66% reduction of capillaries (1–3 capillaries)	

Reference	Points	Age ⩽ 40 years	Age > 40 years

Hoerth et al. [29]	0	>7.75	>8.5
	1	>7.25–7.75	>7.75–8.5
	2	>6–7.25	>6–7.75
	3	⩽6	⩽6

**Table 2:** Enlarged capillary scoring system.

Reference	Points	Enlarged capillary
Koenig et al. [3]	0	Normal
	1	Borderline (<2 times the normal diameter)
	2	Definitely enlarged (⩽2 times, but ⩽4 times the normal diameter)
	3	Extremely enlarged (>4 times the normal diameter)

Dinç et al. [63]	0	Absent
	1	Presence of focal enlarged capillaries or frequent presence of apically enlarged capillaries
	2	Frequent presence of enlarged capillaries or presence of giant capillaries

**Table 3:** Dimensions of capillary parameters reported for healthy adults.

Study (year, country)	Number of patients	Capillary length	Intercapillary distance	Loop diameter	Internal diameter	Capillary width	Apex width	Venous limb	Arterial limb	Reference
Lefford and Edwards (1986, England)	18	146.3 ± 54.7	142.2 ± 26.0			39.8 ± 9.6	34.3 ± 8.9		10.9 ± 2.7	[44]
Grassi et al. (1992, Italy)	25	23.3 ± 51.9				35.4 ± 9.4	38.1 ± 9.4	12.0 ± 3.0		[50]
Kabasakal et al., (1996, England)	38	215 ± 40								[54]
Bukhari et al. (1996, England)	10			14	18	36		11	7	[68]
Schiavon et al. (1999, Italy)	26	255 ± 24	118 ± 22							[10]
Chin et al. (1999, Taiwan)	34									[85]
Bhushan et al. (2000, England)	44			15.2 ± 3.5				13.6 ± 2.6	11.3 ± 2.3	[86]
Bukhari et al. (2000, England)	20			17.2		42.7		15.6	13	[69]
Lambova and Müller-Ladner (2013, Bulgaria)	34	197 ± 70						18 ± 1	13 ± 1	[1]
Hofstee et al. (2013, Netherlands)	14			17.40 ± 4.16		45.56 ± 8.79		15.95 ± 4.08	13.60 ± 2.74	[87]
Graceffa et al. (2013, Italy)	30			26.4 ± 5		46.1 ± 10		17.5 ± 3.1	14 ± 3.2	[88]
Ingegnoli et al. (2013, Italy)	100	237	153.5	18	21	41				[14]
Le and Cho (2014, Korea)	25	270 ± 35		10 ± 2		32 ± 5				[18]

**Table 4:** Capillary length and width scoring systems.

Reference	Points	Length of capillary loops per mm
Redisch et al. [59] Kabasakal et al. [54] Ingegnoli et al. [17]	0	Normal (<300 μm)
	1	Slightly elongated
	2	Moderately elongated
	3	Markedly elongated

Reference	Points	Capillary width per mm

Lee et al. [89] Ingegnoli et al. [17]	0	Normal (<25 to 50 *μ*m)
	1	Normal (<25 to 50 μm)
	2	Definitely widened (90 to 150 μm)
	3	Giant capillaries (>150 μm)

**Table 5:** Intercapillary distance scoring system.

Reference	Points	Intercapillary distance per mm
Lefford and Edwards [44] Ingegnoli et al. [17] Lee et al. [89]	0	Normal (<110 μm)
	1	Slightly increased
	2	Definitely increased
	3	Markedly increased (>190 μm)

**Table 6:** Scoring systems for avascular areas.

Reference	Points	Avascularity per mm
Cutolo et al. [74]	0	No avascular areas
Terreri et al. [48]	1	Mild (1 to 2 avascular areas)
Kabasakal et al. [54]	2	Moderate loss of capillaries (>2 avascular areas)
Ingegnoli et al. [17]	3	Severe (large and confluent avascular areas)

Anders et al. [73]	1	Less than 1 to 3 capillaries
	2	4 to 6 capillaries
	3	More than 6 capillaries

Hofstee et al. [46]	1	None (loss of 2 or more consecutive capillaries)
	2	Moderate (loss of 2 to 4 consecutive capillaries)
	3	Severe (loss of >4 consecutive capillaries, or >2 areas with loss of >2 capillaries)

**Table 7:** Capillary distribution scoring systems.

Reference	Points	Capillary distribution per mm
Cheng et al. [77]	Stage A	
	0	Regular (100%)
	1	Slight irregularity
	Stage B	
	2	Disarranged (<50%)
	3	Disarranged (>50%)
	Stage C	
	4	Local paucity
	5	Enlarged loop bordering local paucity
	6	Complete paucity

Cutolo et al. [7] Ingegnoli et al. [17]	0	Normal distribution (no changes)
	1	Mild disorganization (>33% alterations/mm)
	2	Moderate, disorganization (33 to 66% alterations/mm)
	3	Severe disorganization (>66% alterations/mm)

Hofstee et al. [46]	0	Normal (if there was a regular or slightly disturbed nailfold pattern)
	1	Moderate
	2	Severe

**Table 8:** Nailfold capillaroscopy patterns scoring system.

Study	Patterns	Description
Ingegnoli et al. [20]	Normal	6–8 capillaries/mm, capillaries length between 200 and 500 *µ*, hairpin-shaped loops arranged in parallel rows, with absence of hemorrhages
	Minor abnormalities	6–8 capillaries/mm, <10% of the total loops can be longer than normal, and <50% can be tortuous loops, arranged in parallel rows, with the absence of hemorrhages
	Major abnormalities	⩽6–8 capillaries/mm, >10% of the total loops can be longer than normal, and >50% can be tortuous, enlarged, meandering, and branched loops, disarranged, with the presence of hemorrhages
	Scleroderma pattern	<6 capillaries/mm, >10% of the total loops can be longer than normal, tortuous, branched, bushy, enlarged, and giant loops, disarranged, with presence of hemorrhages

Pavlov-Dolijanovic et al. [78]	Normal	Typical hair pin structure or minor capillary morphological changes in distribution or size of loops
	Nonspecific	Meandering and crossed capillaries, capillary thinning, linear elongation of the loop, focal distribution of capillary hemorrhages, prominent subpapillary plexus, capillary spasm, nonhomogeneous distribution or size of loops, widening of the afferent, and apical and efferent part of loop
	Scleroderma [74]	*Early*: few enlarged/giant capillaries, few capillary haemorrhages, no evident loss of capillaries, and relatively well-preserved capillary distribution
		*Active*: frequent giant capillaries, frequent capillary haemorrhages, moderate loss of capillaries, mild disorganisation of the capillary architecture, and absent or mild ramified capillaries
		*Late*: irregular enlargement of the capillaries, few or absent giant capillaries and haemorrhages, disorganisation of the normal capillary array, severe loss of capillaries with extensive avascular areas, and ramified/bushy capillaries

**Table 9:** Scoring system for subpapillary venous plexus visibility.

Reference	Points	Subpapillary plexus visualization
Wertheimer and Werthelmer [84] Terreri et al. [48]	0	Absence of plexus visibility; capillaries only are seen
	1	Doubtful visibility or the occasional presence of a venule parallel to the nailfold
	2	Plexus visible only in restricted areas or throughout the width of the finger but only close to the distal row of capillaries
	3	Plexus visible throughout the width of the finger but not extending proximally, or if extending proximally, not generalized throughout the width of the finger
	4	Plexus visible throughout the width of the finger and proximally

Kabasakal et al. [54] Ingegnoli et al. [17]	0	Not visible
	1	Doubtful visibility
	2	Plexus visible only in restricted areas
	3	Prominently visible over a wide area
